# Predicting the Social-Emotional Competence Based on Childhood Trauma, Internalized Shame, Disability/Shame Scheme, Cognitive Flexibility, Distress Tolerance and Alexithymia in an Iranian Sample Using Bayesian Regression

**DOI:** 10.1007/s40653-022-00501-1

**Published:** 2022-11-29

**Authors:** Hojjatollah Farahani, Parviz Azadfallah, Peter Watson, Kowsar Qaderi, Atena Pasha, Faezeh Dirmina, Forough Esrafilian, Behnoosh Koulaie, Nazanin Fayazi, Nasrin Sepehrnia, Arezoo Esfandiary, Fatemeh Najafi Abbasi, Kazhal Rashidi

**Affiliations:** 1 Tarbiat Modares University, Tehran, Iran; 2 Cambridge University, Cambridge, UK; 3 Kermanshah University, Kermanshah, Iran; 4 University of Tehran, Tehran, Iran; 5 Azad University, Science and Research, Tehran, Iran; 6 Azad University of Karaj, Karaj, Iran; 7 Imam Hospital, Tehran, Iran; 8 Islamic Azad University, Rudehen, Iran

**Keywords:** Alexithymia, Bayesian models, Childhood trauma, Cognitive flexibility, Disability-shame Scheme, Distress tolerance

## Abstract

The purpose of this study was to predict Social Emotional Competence based on childhood trauma, internalized shame, disability/shame scheme, cognitive flexibility, distress tolerance, and alexithymia in an Iranian sample using Bayesian regression. The participants in this research were a sample of 326 (85.3% female and 14.7% male) people living in Tehran in 2021 who were selected by convenience sampling through online platforms. The survey assessments included demographic characteristics (age and gender), presence of childhood trauma, social-emotional competence, internalized shame, the Toronto Alexithymia scales, Young's measure of disability/shame together with measures of cognitive flexibility and distress tolerance. The results from Bayesian regression and Bayesian Model Averaging (BMA) indicated that internalized shame, cognitive flexibility and distress tolerance can be predictive of Social Emotional Competence. These results suggested that Social Emotional Competence can be explained by some important personality factors.

## Introduction

Social interaction is one of the basic needs of human existence. Since childhood, people constantly seek to identify the mental states and feelings of those around them to determine how to adapt their thinking and behavior to them (Beauchamp & Anderson, 2010; Blakemore, 2010; Frith & Frith, 2012). Social Emotional Competence refers to the ability to care for others, comfort and help them, cooperate and use negotiation, accept compromise and derive pleasure from their achievements (Luteijn et al., 2000). Social Emotional Competence is important to build and maintain relationships, communicate with others, and participate in social interactions (Cacioppo, 2002). These skills develop gradually throughout childhood and adolescence.

Sufficient Social Emotional Competence requires a complex interaction between cognitive, behavioral, neurological, and environmental factors (Frith & Frith, 2012; Soto-Icaza et al., 2015). Individual differences indicate that the impact of life traumatic events on distress outcomes is not direct (Verhaeghe & Vanheule, 2005) but through a variety of factors.

According to the latest research childhood trauma is a direct predictor of decreased Social Emotional Competence in childhood and adolescence (Alink et al., 2012; Kim & Cicchetti, 2004; Kinard, 1999; Levendosky et al., 1995). Childhood trauma appears in the forms of physical, sexual, and emotional abuse and epidemiological studies have shown a strong association between childhood trauma and general mental health problems (e.g., Allen, 2008; Anda et al., 2002; Chapman et al., 2004; Edwards et al., 2003; Putnam, 2003). Additionally, the World Health Organization (2019) claims that childhood trauma is a pervasive global problem that is estimated to be experienced by one billion children between the ages of two and seventeen. 20% of women and 10% of men have experienced sexual abuse and 20% of children report physical abuse (Naghavi et al., 2011). Furthermore, people with traumatic experiences in childhood are more likely to feel lonely and experience social avoidance, and self-blame (Arseneault et al., 2010; Graham & Juvonen, 1998; Olweus, 1993; Schacter et al., 2015). They experience more rejection and less acceptance by their peers (Cullerton-Sen & Crick, 2005; Veenstra et al., 2007). Not surprisingly, they may have difficulty forming new social relationships during the transition to adulthood and may feel lonelier, have less social support, are less likely to have a cohabiting partner, and have poorer family functioning in adulthood (Day et al., 2017; Sigurdson et al., 2014). As a result, adverse childhood traumatic experiences are associated with shame (McLean & Foa, 2017), accompanying feelings of worthlessness, inferiority, and incompetence resulting from failure (Tangney et al., 2011; Wong & Tsai, 2007).

Inner shame is a degrading emotional experience that is associated with avoidance tendencies, negative self-esteem, and feelings of self-control (such as self-loathing) that can affect an individual's cognition, perception, feelings, and functions (Gilbert & Miles, 2000; Gilbert et al., 2010; Matos et al., 2013; Michail & Birchwood, 2013; Moitra et al., 2008). Zimbardo (2003) believes that shame is an extreme inhibitor in interpersonal relationships making people afraid of expressing themselves and being very sensitive about others’ reactions leading quickly to frustration. There is, consequently, considerable research on the impact of family environment on emotional development (e.g., Gottman et al., 1997; Gross, 1999; Sroufe, 1996). Studies of clinical (Berenbaum, 1996; Zlotnick et al., 2001) and non-clinical samples (Clayton, 1997; Turner & Paivio, 2002) have shown that both physical and sexual abuse of children predict impaired consciousness and emotional expression.

Alexithymia has been described as a personality trait that includes difficulty identifying and describing emotions, poorly developed thinking, and lack of imagination (Sifneos, 1973). Alexithymia is often negatively associated with physical and mental well-being (Taylor et al., 1999). More specifically, a higher rate of alexithymia was found in people with a history of childhood abuse (Berenbaum, 1996; Berenbaum & James, 1994; Bermond et al., 2008; Kench & Irwin, 2000). Unresolved and unprocessed emotions can lead to subsequent physiological disorders of nervous systems, health problems, and a wide range of symptoms of psychological distress (e.g., Karukivi et al., 2010; Marchini et al., 2018; Martino et al., 2021; Pennebaker & Beall, 1986).

Cognitive flexibility, on the other hand, is a range of human abilities in the field of recognizing and adapting to the environment to change behavioral strategies (Khalajzadeh & Hashemi, 2019) that allow us to adapt and express our thoughts and behaviors quickly in response to changing environmental demands and goals (Chevalier et al., 2012; Miyake et al., 2000). Cognitive flexibility enables children to control core internal thoughts and change external behavior in order to recognize, label, interpret and respond to their own and others' emotions (Silkenbeumer et al., 2016). People who have the ability to think flexibly are more psychologically resilient than people who are not flexible thinkers (Phillips, 2011). Simons and Gaher (2005) defined distress tolerance as a self-perceived ability of a person to experience and tolerate negative emotional states or behavioral ability to persist in goal-oriented behavior when experiencing emotional distress. As a result, low levels of distress tolerance create a kind of emotion dysregulation in the individual that leads to maladaptive behavioral responses and stressful situations (Keough et al., 2010).

Overall, according to the previous research, childhood trauma is associated with negative physical health problems, short-term and long-term psychological disorders (Cook et al., 2005; Lueger-Schuster et al., 2018), long-term interpersonal problems (Rumstein-McKean & Hunsley, 2001; Wilson & Scarpa, 2015) and feelings of shame (Bockers et al., 2016), and distress (Bower et al., 2014; Gouin et al., 2012; Hazel et al., 2008; Morris et al., 2017; Turner & Lloyd, 1995; Turner & Turner, 2005). However, not all victims of trauma react in the same way. Some may experience dysfunction but gradually return to normal, while others develop clinically significant mental disorders (Smyth et al., 2008), such as depression (Briere, 2004; Hill, 2003) and anxiety (Heim & Nemeroff, 2001) that require long-term intervention (Bonanno & Mancini, 2012; Espié et al., 2009; Kessler et al., 2005).

Finally, some models examine the relationship between Social Emotional Competence and its determinants. Most of these models are only theoretical and rarely comprehensively tested to provide explanations of the factors that predict Social Emotional Competence. Therefore, to obtain a better insight into Social Emotional Competence and its relationship with childhood traumatic experiences, in the present study we aimed to predict Social Emotional Competence based on childhood trauma, shame, alexithymia, cognitive flexibility, and distress tolerance. We assume that childhood trauma will predict social emotional competence. Our second hypothesis is that shame will predict social emotional competence. Further, we suggest that alexithymia will predict social emotional competence. Our fourth hypothesis is that cognitive flexibility will predict social emotional competence. Lastly, we hypothesise that distress tolerance will predict social emotional competence.

## Method

### Participants

The participants in this research were a sample of 326 people living in Tehran in 2021 who were selected by convenience sampling through online platforms. The survey included the Childhood Trauma Questionnaire (CTQ), the Social Emotional Competence questionnaire, the Internalized Shame Scale, Young's Disability/Shame Scheme Questionnaire, the Cognitive Flexibility Questionnaire (CFI), the Distress Tolerance Scale, the Toronto Alexithymia Scale and demographic characteristics (age and gender). Answering all items was mandatory, therefore, there was no missing data reported.

### Demographic Characteristics

The average age of the 326 participants was 32.2 (SD = 12.4) with 85.3% (277) of participants being female (mean age = 29.4, sd- = 14.2) and 14.7% (48) of participants being male (mean age = 37.8, sd = 12.7).

## Measures

### Childhood Trauma Questionnaire (CTQ)

The Childhood Trauma Questionnaire (CTQ) was developed by Bernstein et al. (2003) to assess childhood traumatic experiences. This questionnaire is a screening tool to identify people with experiences of childhood abuse and neglect. This questionnaire can be used for both adults and adolescents. The scale contains five subscales, each with five items: Emotional Abuse, Physical Abuse, Sexual Abuse, Emotional Neglect, and Physical Neglect. Items are responded to using a 5-point scale ranging from "Never true" (0) to "Very often true" (4), and summed scores for the subscales have a possible range of scores from 0 to 20, with higher scores suggesting more severe maltreatment. The questionnaire has 28 questions, 25 of them are used to assess the main components of the questionnaire and 3 of them are used to identify people who deny their childhood problems. In the study of Bernstein et al. (2003) Cronbach's alpha coefficients of the questionnaire on a group of adolescents on the dimensions of emotional abuse, physical abuse, sexual abuse, emotional neglect, and physical neglect were equal to 0.87, 0.86, 0.95, 0.89 and 0.78 respectively. Further, its concurrent validity with therapists' rating of childhood traumatic experiences rates has been reported in the range of 0.59 to 0.78 (Bernstein et al., 2003). In Iran, Ebrahimi et al. (2013) have reported this questionnaire’s Cronbach's alpha as ranging from 0.81 to 0.98 for its five components. Cronbach's alpha in this research ranged from 0.83–0.89.

### Social Emotional Competence questionnaire

The Social Emotional Competence questionnaire, designed and developed by Zhou and Ee in 2012 to measure students’ Social Emotional Competence, includes five dimensions: self-awareness, social awareness, self-management, relationship management, and responsible decision-making. It has 25 items (such as “if someone is sad, angry or happy, I know what they are thinking.”) each with a six-point Likert scale ranging from fully disagree (1) to fully agree (6). Emamgholivand et al. (2019), in their study, evaluated the content, face, and criterion validity of this questionnaire as appropriate. Cronbach's alpha coefficient calculated in the study of Emamgholivand et al. (2019) for this questionnaire was estimated above 0.7. Cronbach's alpha in this research is 0.76.

### Internalized Shame Scale

Cook's Internalized Shame Scale was developed in 1993 and includes 30 items and two subscales of shyness and self-esteem. Each item on this scale consists of a five point Likert scale (never = 0, very little = 1, sometimes = 2, often = 3, always = 4). High scores on this scale indicate worthlessness, inadequacy, feelings of inferiority, emptiness, and loneliness. A low score indicates high self-confidence. In Cook (1993) Cronbach's alpha reliability coefficients of shyness and self-esteem subscales of internalized shame scale were 0.94 and 0.90, respectively. In Rajabi and Abbasi (2011) Cronbach's alpha reliability coefficients for the internalized shame scale were 0.90 in the whole sample, 0.89 in men and 0.91 in women. Cronbach's alpha in this research ranged from 0.88–0.91.

### Young's Disability/Shame Scheme Questionnaire

This scale assessed 15 maladaptive schema in 75 items and was developed by Waller et al. (2001). The Persian version of this scale was validated by Sadoughi et al. in 2008 in an Iranian student sample. In this paper only the score of the Disability/Shame Scheme subscale of the scale was analyzed. This questionnaire has ten items and takes the form of closed-ended questions each with a six point Likert scale ranging from fully wrong (1) to fully correct (6). The confirmatory analysis in this research indicated this scale has been explained by one factor accounting for 65.73 percent of item variance. Fitness indices are acceptable (GFI = 0.89, CFI = 0.9, RMSEA = 0.07). Cronbach's alpha is 0.93 in this research.

### Cognitive Flexibility Questionnaire (CFI)

The Cognitive Flexibility Questionnaire is a brief 20-item self-report instrument developed by Dennis and Vander Wal (2010) to measure cognitive flexibility that is needed in a person's situation to challenge and replace dysfunctional thoughts with more efficient ones. Its scoring method is based on a five point Likert scale ranging from strongly disagree (1) to strongly agree (5) and tries to measure three aspects of cognitive flexibility: a) the desire to understand difficult situations as controllable situations; b) the ability to understand multiple alternatives to life events and human behavior; and c) the ability to create multiple alternative solutions to difficult situations. This questionnaire is used in clinical and non-clinical settings to assess the individual's progress in developing flexible thinking in cognitive-behavioral therapy of mental illness (Dennis & Vander Wal, 2010). Its convergent validity with the Martin and Robin Cognitive Flexibility Scale was 0.75. Cronbach's alpha coefficient was reported as 0.90 for the whole scale and 0.87, 0.89, and 0.55 for the subscales, respectively. CFI also has appropriate convergent and concurrent factor validity in Iran. Unlike the main scale where two factors were obtained, In the Persian version the cognitive flexibility questionnaire has three factors: perception of controllability, perception of different options, and perception of justification of behavior (Kohandani & Abolmaali Alhosseini, 2017). Cronbach's alpha in this research ranged from 0.81–0.87.

### Distress Tolerance Scale

This scale is an emotional distress self-assessment questionnaire developed by Simons and Gaher in 2005. Items on this scale assess anxiety tolerance based on the individual's abilities to cope with emotional distress, subjective assessment of distress, attention to negative emotions, if they occur, and regulatory actions to cope with distress. This scale includes 15 questions and four subscales of emotional distress tolerance, absorption by negative emotions, mental evaluation of distress, and adjustment of efforts to reduce distress. Items on this scale are scored on a five point Likert scale from strongly disagree (1) to strongly agree (5).

High scores on this scale indicate high distress tolerance. The alpha coefficients for these subscales are 0.72, 0.82, 0.78, 0.70 respectively and for the whole scale is 0.82. Azizi et al. (2010) reported a Cronbach's alpha value of this questionnaire as 0.67 and the validity of the retest of this questionnaire as 0.79. Cronbach's alpha in this research ranged from 0.82–0.88.

### The Toronto Alexithymia Scale

The Persian version of the Toronto Alexithymia Scale (Bagby et al., 1994) is a 20-item test on a five point Likert scale ranging from 1 (completely disagree) to 5 (completely agree). There are three subscales of difficulty identifying feelings (DIF), difficulty describing feelings (DDF), and externally oriented thinking (EOT). A total score is calculated from the sum of the scores of the three subscales for total alexithymia. The internal consistency of each of the subscales of this questionnaire in terms of Cronbach's alpha is equal to 0.83, 0.77, and 0.73, respectively, and Cronbach's alpha for the whole scale is equal to 0.82 (Tull et al., 2005). Cronbach's alpha of this scale was 0.85 in the Iranian sample for the whole scale and 0.82, 0.75, and 0.72 for the above subscales, respectively (Besharat, 2007). Cronbach's alpha in this research ranged from 0.79–0.83.

### Statistical Analysis

Bayesian statistical methods are emerging as a preferred tool among a vast spectrum of psychological research. To briefly explain, Bayes’ Theorem is a model that gives information about the data, and eventually, the Bayesian paradigm interprets probability as a mental experience of uncertainty. Bayesian methods are not designed to determine the falsity of the null hypothesis but rather aim to capture the strength of the evidence for the hypothesized opinion.

Consequently, Bayesian Analysis formulates a probability distribution for the parameters and their mathematical functions which is a combination of the Likelihood distribution and the prior distribution as a representation of the researcher’s initial knowledge.

Kruschke (2015) presents two main ways which are used to give credible regions to show the distribution of the posterior density of an estimated parameter for each variable. The highest density interval (HDI) produces an interval containing values which have the highest probability density and always contains the most likely value of the parameter value corresponding to the mode. The equal-tailed interval (ETI), on the other hand, is an interval where the probability of being below the interval is as likely as being above it. This interval will include the median. These two credible regions agree if the posterior distribution of a parameter is symmetric but will differ if it is skewed. In the current research, the 95% credible interval is calculated with the Highest Density Interval (HDI), and the Equal Tailed Interval (ETI). The total of the posterior probabilities for values inside these intervals is 0.95.

We also evaluated the Mean, and Median of each variable’s posterior distribution for the univariate regression coefficients of each variable with Social Emotional Competence. We also give the value of the parameter with the maximum posterior probability (MAP), ie the most likely value of the parameter.

In order to determine the role of each predictive variable in emotional competence the five Childhood Trauma subscales (emotional abuse, physical abuse, sexual abuse, emotional neglect, and physical neglect) together with Internalized Shame, Disability/Shame Scheme, Cognitive Flexibility, Distress Tolerance and Alexithymia were used as predictors in a Bayesian regression. In addition, to measure the relative effect of each variable, Bayesian Model Averaging (BMA) was used in which the weighted fitted values resulting from various methods are combined to evaluate the posterior distribution of that model instead of relying on a single model as the ‘true’ model. BMA, therefore, presents the most plausible models having the highest posterior probabilities of reproducing the relationships in the data, in our case predicting social emotional competence, and allows a comparative assessment of their relative importance which is a more realistic approach to modelling (Hinne et al., 2020). Bayesian inference criteria (BIC) are also presented which combine the degree of fit of the model to social emotional competence with the complexity of the model with more accurate, more parsimonious models being preferred. The lower the BIC, therefore, the better the model.

We also evaluate p! = 0 to determine the presence of a certain variable in the regression models and, hence, to assess the chance of each scale being related to social emotional competence. We are, therefore, able to express the relative importance of different variables across possible different models.

## Results

Table 1 shows the mean, range and the standard deviation of the studied variables.

**Table 1:** The mean, standard deviation and range of the variables (*n* = 326)

Variable	M	SD	RANGE
Internalized shame	78.1	13.7	51–118
Disability/Shame scheme	17.9	8.1	9–49
Cognitive flexibility	70.9	9.4	15–91
Distress tolerance	43.3	10.5	18–75
Alexithymia	48.5	10.5	27–85
Social emotional competence	73.9	9.9	37–95
Childhood trauma total	41.3	13.2	27–104
Affective abuse	8.0	3.7	5–24
Physical abuse	6.6	3.0	5–23
Sexual abuse	7.2	3.6	5–24
Affective neglect	12.0	3.6	6–25
Physical neglect	7.6	2.9	5–19

As can be seen in Table 2, internalized shame, cognitive flexibility and distress tolerance, though none of the five childhood trauma scales, are predictive of Social Emotional Competence with 95% credibility.

**Table 2:** The 95% credible intervals (CIs) using highest density and equal tailed methods

Parameter	HDI		ETI	
Intercept	40.99	66.54	41.46	67.10
Internalized shame	-0.34	-0.13	-0.33	-0.12
Disability/Shame scheme	-0.08	0.25	-0.08	0.26
Alexithymia	-0.17	0.06	-0.17	0.06
Cognitive flexibility	0.34	0.52	0.33	0.52
Distress tolerance	0.10	0.29	0.10	0.29
Affective abuse	-0.67	0.07	-0.66	0.09
Physical abuse	-0.01	0.80	-0.03	0.78
Sexual abuse	-0.20	0.35	-0.20	0.35
Affective neglect	-0.30	0.34	-0.29	0.34
Physical neglect	-0.48	0.33	-0.48	0.32

Table 3 gives the Map, Mean, and Median of the variables’ posterior distribution for the univariate regression coefficients of each variable with Social Emotional Competence. We see that the highest posterior regression coefficients in Table 3 back up the results in Table 2 by clearly showing that internalized shame, cognitive flexibility and distress tolerance have the highest magnitudes of relationships with Social Emotional Competence.

**Table 3:** Posterior distribution summary measures of regression coefficients showing the degree of univariate associations with Social Emotional Competence: Maximum Probability (Map), Mean, and Median

Variable	Map	Mean	Median
Internalized shame	-0.22	-0.23	-0.23
Disability/Shame scheme	0.00	0.09	0.08
Alexithymia	-0.00	-0.06	-0.06
Cognitive flexibility	0.42	0.42	0.42
Distress tolerance	0.22	0.20	0.19
Affective abuse	-0.00	-0.29	-0.29
Physical abuse	0.01	0.38	0.38
Sexual abuse	0.00	0.07	0.07
Affective neglect	-0.00	0.02	0.02
Physical neglect	-0.00	-0.08	-0.08

BMA was used to calculate each variable’s relative importance. As can be seen from Table 4, the 5 remaining models share almost the same BIC which indicates that these competitive models are only slightly different from each other. However, the smallest value for BIC shows the posterior probability as 0.67 which means there is still uncertainty regarding the model, therefore, finding the average of all the models could be a way to consider this uncertainty. It is worth mentioning that if only one model was to be selected, this uncertainty in the model could not be justified. The EV column shows the average of model coefficients.

**Table 4:** 5 models with highest posterior probabilities to predict social emotional competence (cumulative posterior probability = 1) resulting from Bayesian Model Averaging

Variable	p! = 0	EV	SD	Model 1	Model 2	Model 3	Model 4	Model 5
Intercept	100.0	51.52	5.94	51.45	51.24	51.68	51.51	51.55
IS	100.0	-0.22	0.04	-0.22	-0.23	-0.24	-0.24	-0.21
SYO	5.0	0.00	0.03	-	-	0.06	-	-
AL	4.3	-0.00	0.01	-	-	-	-	-
COG	100.0	0.42	0.05	0.42	0.42	0.43	0.42	0.42
DIS	100.0	0.22	0.05	0.22	0.22	0.22	0.21	0.21
AA	4.4	-0.00	0.03	-	-	-	-	-0.07
PA	6.6	0.01	0.05	-	0.16	-	-	-
SA	4.9	0.00	0.03	-	-	-	0.09	-
AN	3.7	-0.00	0.02	-	-	-	-	-
PN	3.8	-0.00	0.03	-	-	-	-	-
r2				0.43	0.43	0.43	0.43	0.43
BIC				-164.00	-159.37	-158.81	-158.76	-158.54
Post prob				0.67	0.07	0.05	0.05	0.04

p! = 0 is the posterior probability that the regression coefficient is non-zero expressed as a percentage. EV is the regression estimate for each variable formed by a weighted averaging of the regression coefficients from the five best fitting models with the weights representing the posterior probability for that model. Similarly SD is the weighted average of the standard deviations from the five best fitting models

*IS* Internalized Shame, *SYO* Disability/Shame Scheme, *AL* Alexithymia, *COG* Cognitive Flexibility, *DIS* Distress tolerance, *AA* Affective Abuse, *PA* Physical Abuse, *SA* Sexual Abuse, *AN* Affective Neglect, *PN* Physical Neglect

Looking at the values of the p! = 0 column in Table 4, internalized shame, cognitive flexibility and distress tolerance have a 100% chance of being related to social emotional competence when looking across all the likeliest occurring models. None of the other scales have more than a 10% chance of being related to social emotional competence.

**Fig. 1:**
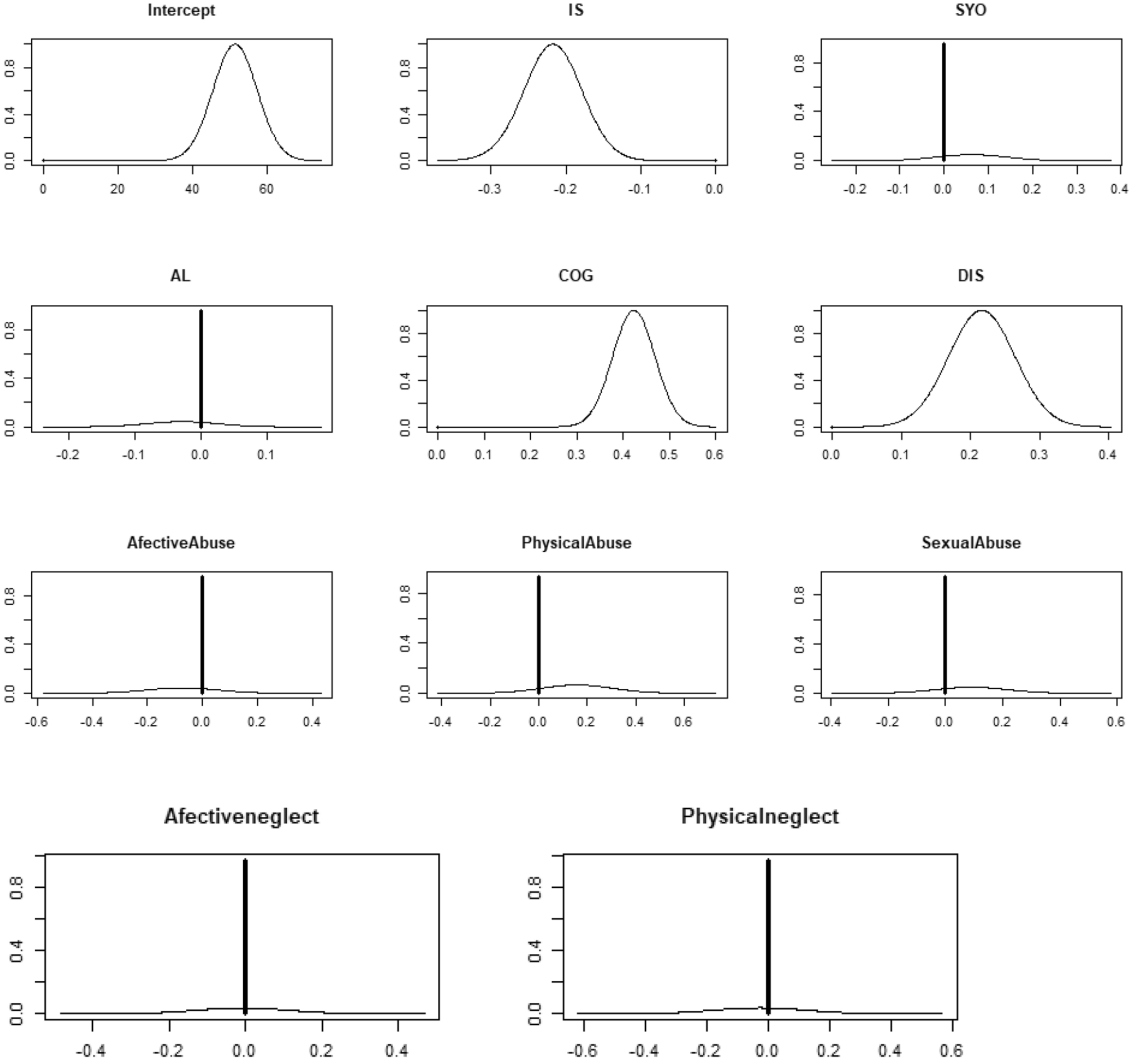
The posterior distributions of the regression coefficients presented in Table 4 for predicting social emotional competence. Zero values of the regression coefficients are shown by a solid line and the respective heights on the y-axis show the posterior probabilities of the regression coefficient taking a value on the x-axis

Figure 1 demonstrates the posterior distribution of the regression coefficients in the models reported in Table 4. Zero values of the regression coefficients are shown by a solid line and the respective heights on the y-axis show the posterior probabilities of the regression coefficient taking a value on the x-axis. Internalized shame, cognitive flexibility and distress tolerance all show peaks away from zero.

## Discussion

This study aimed to predict Social Emotional Competence based on childhood trauma, shame, alexithymia, cognitive flexibility and distress tolerance. The results from the analyses in this paper show that there is a clear positive and significant relationship between Social Emotional Competence and internalized shame, cognitive flexibility, and distress tolerance. These three scales all individually show relationships with Social Emotional Competence with non-zero 95% credible regions (Table 2), univariate regression coefficients with values above 0.2 in absolute value (Table 3) and a 100% chance of being associated with Social Emotional Competence when pooling information from the Bayesian multiple regressions as shown in Table 4 with associated peaks of posterior distributions of these regression estimates away from zero as shown in Fig. 1. These results are in line with the findings of Wang et al. (2021), Hill et al. (2018), Hashemi and Mashkooraftehi (2020), Jones et al. (1986), Yuen (2019), Zimbardo (2003), Moitra et al. (2008), Michail and Birchwood (2013), Gilbert et al. (2010), Matos et al. (2013).

To explain the results, it can be stated that one of the roots of negative thinking is the feeling of unworthiness where people believe that they are not good enough (Hamidi et al., 2017). Social competence indicates the ability to establish relationships with peers (Hashemi & Mashkooraftahi, 2020). Individual’s beliefs and attitudes are formed under the influence of family and culture, and people who are brought up under strict beliefs also act more effectively and efficiently in society. Based on the model of effective social competence presented by Draganidis and Mentzas (2006), effective message sending, effective message receiving and experimentation were introduced as three important elements of effective social competence. It follows that knowledge of non-verbal communication helps a person to act effectively in sending, receiving, reading, and interpreting non-verbal messages and plays an important role in building intimacy and trust in establishing mutual understanding and social adjustment. In general, interpreting and understanding non-verbal behaviors facilitates social interactions through the understanding of others' emotional states. Evidence suggests that interaction with peers can be considered as an important factor in a person's social network, although other factors in the network, including relationships with parents and other family members and present and previous experiences are influential in shaping the perception of self-esteem and values (Safar Hamidi, 2016). Inappropriate parenting styles, childhood traumas, wrong patterns and behaviors, and improper presence or absence of parents can lead to behavioral, emotional, and cognitive problems and disruption in a child/adolescent’s psychological development (Khalajzadeh & Hashemi, 2019).

The first finding showed that internalized shame is a positive predictor of Social Emotional Competence, which is consistent with the studies of Lewis and Sullivan (2005), Lewis (1971), Lewis (1995), Schlenker (1980), Yasini Farid et al. (2013), Jones et al. (1986). To explain this finding, one of the psychological constructs related to Social Emotional Competence is internalized shame. Conscious emotions play an important role in children's motivation, social competence, and adjustment (Lewis & Sullivan, 2005). Among conscious emotions, shame needs to be further considered. All human beings face events in their daily lives in which they experience feelings of failure, imperfection, and inadequacy. Following these experiences, evaluations of emotions are formed in human beings. Shame is a self-conscious emotion that is evoked by self-reflection and self-evaluation (Tangney, 2003). Internalized shame is a degrading emotional experience that is associated with avoidance tendencies, negative self-esteem, and feelings of self-control (such as self-loathing) and it can affect a person's cognition and feelings in self-perception and individual functions. Individuals who have negative self-perceptions evaluate themselves with a lack of confidence and a consequence of this evaluation is feeling internalized shame (Gilbert & Miles, 2000; Gilbert et al., 2010; Matos et al., 2013; Moitra et al., 2008). Zimbardo (2003) believes that shame is an extreme precaution in interpersonal relationships and causes people to be afraid to express themselves, to be very sensitive about others’ reactions, and to become frustrated quickly. In addition, feelings of shame cause social anxiety, lack of self-confidence, lack of social skills, and incompatibility with groups (Yasini Farid et al., 2013). Schlenker's (1980) study showed that being concerned about others’ inappropriate evaluation can be seen as an inhibitory factor in the context of socio-psychological phenomena such as self-assertiveness, social supportive behavior, self-defeating and social anxiety.

The research also revealed that cognitive flexibility is a positive predictor of Social Emotional Competence, which is in line with the findings of Wang et al. (2021), Hill et al. (2018), Hashemi and Mashkooraftahi (2020), Yuen (2019), Kashdan and Rottenberg (2010), Moses and Carlson (2004), Silkenbeumer et al. (2016). Alexithymia includes a wide range of human abilities from the ability to recognize and cope with the environment to changing behavioral strategies (Khalajzadeh & Hashemi, 2019). The theory of learned helplessness (Seligman, 1972) refers to a situation in which a person, based on past experiences (such as persistent failures), believes that they will not succeed in whatever they do, therefore they give up trying (Nolen-Hoeksema et al., 1986). According to this theory, the low level of flexibility in thoughts and beliefs causes the generalization of misconceptions to different life situations and makes a person prone to some mental disorders such as depression. Cognitive flexibility encompasses a wide range of individual abilities where one can identify different situations and adapt to them (Klohnen, 1996). Given that stressful situations are emotional, a person's degree of flexibility plays an important role in how one copes. It follows that people with low flexibility in the face of unpleasant experiences, instead of perceiving a stressful situation as a controllable situation, follow an inflexible pattern in adapting to the situation (Lougheed & Hollenstein, 2016). When this inflexible pattern in a person's performance continues, it leads to a decrease in the individual's capacity to tolerate unpleasant experiences (Gündüz, 2013). It follows that the person, by distancing himself and through inattention, tries to escape from the stressful situation and very soon feels failure, worthlessness, and helplessness (Kertz & Woodruff-Borden, 2013). People who have experienced childhood trauma are prone to low resilience due to growing up in unsafe environments, which makes them less resilient in the face of stressful situations (Dickstein et al., 2007). Moses and Carlson (2004) believed that children should develop their abilities both to adapt to a changing environment and to think about their thoughts and behaviors in order to perceive their own and others' perspectives on the world. The period of 3 to 5 years is a key period for the development of cognitive flexibility (Wang et al., 2021). Cognitive flexibility enables children to control core internal thoughts and change external behavior in order to recognize, label, interpret and respond to their own and others' emotions (Silkenbeumer et al., 2016). The early attainment of cognitive flexibility provides children with greater exposure and opportunities to apply cognitive skills to facilitate their subsequent development of more comprehensive skills (e.g., self-monitoring and remedial approaches), which promotes increased performance in emotion understanding (Oakhill & Cain, 2012). This is influential in the development of subsequent emotional perception. Improving cognitive flexibility is associated with greater competence in emotion perception (Wang et al., 2021). The quality of parental and child care, and the quality of childhood experiences in general, is associated with cognitive flexibility (Gündüz, 2013). Yuen (2019) noticed that there is a significant relationship between individuals' perception of competence and flexibility. Flexibly minded people do not avoid stress in their lives, instead, they consider stressful situations as an opportunity for their growth and development. The more flexible a person is the more difficult situations he can consider as controllable situations. In the face of life events and difficult situations, one can think of alternative solutions, and therefore his capacity to cope with stress increases (Hashemi & Mushkooraftahi, 2020).

The results also showed that distress tolerance is a positive predictor of Social Emotional Competence that is in line with the findings of Kashdan and Rottenberg (2010), Rezapour Mirsaleh and Esmaeelbeigi Mahani (2017), Simons and Gaher (2005). Social Emotional Competence refers to the ability and skill to deal with interpersonal interactions and to regulate emotional experiences to achieve desirable outcomes in emotional situations (Sharma, 2012). In addition, there is the ability to regulate emotional response in the definition of Social Emotional Competence and this includes knowing how a person controls his motivations and regulates his emotions in response to others’ reactions and emotions (Monnier, 2015). According to Schmidt-Fajlik (2013), some skills that build Social Emotional Competence include being aware of one's emotional states, successfully expressing one's emotions, interpreting others' emotions and empathy and sympathy with others. Studies showed that cognitive flexibility plays an important role in a wide range of disorders such as anxiety, stress, and distress (Masuda et al., 2014). Studies showed that with increasing cognitive flexibility in people with experience of childhood trauma, distress tolerance increases (Kashdan & Rottenberg, 2010; Rezapour Mirsaleh & Esmaeelbeigi Mahani, 2017). Trafton and Gifford (2011) stated that distress tolerance can be defined as the ability not to respond to the opportunity for immediate negative reinforcement in distressing situations which manifests itself in two forms. One form refers to a person's ability to tolerate negative emotions, and the other form is behavioral manifestations of enduring unpleasant internalized states, which are called smooth loneliness in different situations (O’Cleirigh et al., 2007). Low levels of distress tolerance create a kind of emotional dysregulation in the individual that leads to maladaptive behavioral responses and stressful situations thus people who have a high tolerance for distress are less resilient to negative events caused by the emotional pressures of problems, and often consider stressful situations to be transient (Keough et al., 2010).

The research also revealed that childhood trauma, disability/shame scheme, and alexithymia are not positive predictors of Social Emotional Competence, which is in line with the findings of Salami et al. (2017), Miller-Graff et al. (2017), Sajadi et al. (2015).

To address this, researchers typically provide consistent information about problems resulting from childhood trauma when children are seriously injured or clearly unwell, but in nonclinical samples, in assessments of individuals' performance, Social-Emotional Competence shows different results (Miller-Graff et al., 2017). Past research indicates that childhood trauma, disability/shame schema, and alexithymia can be considered as important factors in a person's network of social relationships and social emotional competence, however, in shaping perceptions of one's own competence and worth, other factors in this network, including internalized shame, cognitive flexibility and distress tolerance, have shown more impact (Mays et al., 1998). It has been found that the deficiency in social competence plays a major role in the etiology of the difficulties of people with childhood trauma and causes these people to become vulnerable to external pressures (social environment) and internal pressures (cognitive conflicts) and causes psychological, social and behavioral problems for them (Salemi et al., 2017). There is considerable evidence that children not only directly benefit from social support networks within the family, but support networks outside the home may also be important, particularly for younger children who spend a lot of time with their caregivers. Child maltreatment is a strong predictor of reduced social competence in childhood and adolescence, whereas social support has been a significant enhancing factor in a child's social competence with higher levels of a child's social competence being reported when caregivers have given more social support (Salami et al., 2017).

On the other hand, shame is one of the psychological constructs related to social anxiety. The study by Stockton et al. (2011) shows that people's concern about social-emotional competence is an intra-individual inhibiting factor that is reflected in the context of social psychological phenomena such as self-expression, social support behavior, and social anxiety. In other words, the experience of shame in many people is not related to their social-emotional competence but these people have very high standards for themselves and often experience shame by comparing their achievements with others. Therefore, the way people evaluate their performance may influence their view of their social competence.

Further, the review of previous literature shows that the expression of emotion is an essential factor in determining health and having a successful performance in social interactions (Thompson, 1994). Childhood traumas affect physiological, psychological, and social development, information processing, children's ability to regulate physiological arousal and loss of self-regulation, as well as the formation of alexithymia (Porche et al., 2011). People's responses to trauma, however, will vary and the variables of family socioeconomic conditions, parental support, and parenting style can moderate this relationship (Sajadi et al., 2015).

One of the limitations of this study is the use of self-report tools and participants may tend to present a desirable image of themselves due to conservatism or social desirability. Due to a large number of participants, it was not possible to conduct a clinical interview in parallel with completing the questionnaires. In this regard, a clinical interview is suggested to build trustful cooperation to complete questionnaires. Other psychosocial factors may also be involved in increasing or decreasing the perception of competence in people with childhood trauma, which should be identified and studied in future research. In particular, experimental research is needed to determine how social, cognitive, and behavioral skills training can affect Social Emotional Competence in individuals experiencing childhood trauma. Furthermore, longitudinal studies can be effective in revealing more accurately the causal relationship between Social Emotional Competence, shame, alexithymia, cognitive flexibility, and distress tolerance in people with childhood traumatic experiences.

## Data Availability

Would be made available in a zip folder on the web if paper is published.

## Code Availability

Would be made available in a zip folder on the web if paper is published.
